# Label-free approaches for extracellular vesicle detection

**DOI:** 10.1016/j.isci.2023.108105

**Published:** 2023-09-30

**Authors:** Loredana Leggio, Greta Paternò, Silvia Vivarelli, Aurelio Bonasera, Bruno Pignataro, Nunzio Iraci, Giuseppe Arrabito

**Affiliations:** 1 Department of Biomedical and Biotechnological Sciences, University of Catania, Catania, Italy; 2 Department of Biomedical and Dental Sciences, Morphological and Functional Imaging, Section of Occupational Medicine, University of Messina, Messina, Italy; 3 Department of Physics and Chemistry - Emilio Segrè, University of Palermo, Viale delle Scienze, building 17, 90128 Palermo, Italy

**Keywords:** Molecular spectroscopy techniques, Molecular medicine

## Abstract

Extracellular vesicles (EVs) represent pivotal mediators in cell-to-cell communication. They are lipid-membranous carriers of several biomolecules, which can be produced by almost all cells. In the current *Era* of precision medicine, EVs gained growing attention thanks to their potential in both biomarker discovery and nanotherapeutics applications. However, current technical limitations in isolating and/or detecting EVs restrain their standard use in clinics. This review explores all the state-of-the-art analytical technologies which are currently overcoming these issues. On one end, several innovative optical-, electrical-, and spectroscopy-based detection methods represent advantageous label-free methodologies for faster EV detection. On the other end, microfluidics-based lab-on-a-chip tools support EV purification from low-concentrated samples. Altogether, these technologies will strengthen the routine application of EVs in clinics.

## Graphical abstract

**Figure:**
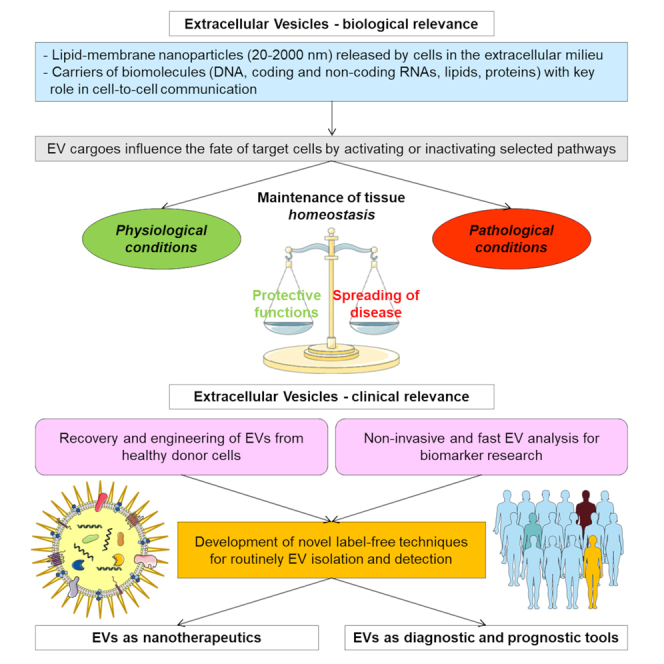

## Introduction

Extracellular vesicles (EVs) are naturally occurring, lipidic-membranous nanocarriers (20–2000 nm) of several macromolecules (such as DNA, RNAs, lipids, proteins) which are produced by almost any typology of cell. The possibility to recover EVs from tissues and biofluids opened a new way for non-invasive research of novel biomarkers.^1^ Accordingly, the detection and analysis of EVs are emerging applications for both diagnosis and therapy. Many studies showed that EVs participate in several pathological processes, such as cancer development and progression, immune response modulation^2^ and neurodegenerative diseases, either as triggers of the disease or as neuroprotective players.^3^^,^^4^^,^^5^ Interestingly, the intrinsic abilities of EVs to deliver different biomolecules with low immunogenicity^6^^,^^7^ and to cross the biological barriers^8^^,^^9^ have been exploited to design EV-based advanced nanotherapeutics.^10^

Several methods have been developed for EV recovery, purification and characterization, considering also the great diversity of molecular cargoes shuttled via EVs.^11^^,^^12^^,^^13^^,^^14^ However, the diagnostic potential of EVs is not fulfilled yet, due to the lack of definitive EV-associated biomarkers.^15^ The current scenario calls for a further effort in terms of fundamental research to fill this gap, using better disease models and larger patient cohorts. In parallel, as in the past for scientific advancement, a new set of technologies is required to fasten the discovery of EV biomarkers and their practical use in the clinical routine. To this end, label-free approaches can open up the way to clinical applications for EVs, given their ideal integration into miniaturized lab-on-chip platforms for EV biomarker detection.^16^

## EVs, where we are now: State of the art and current limitations

Based on their size, EVs are classified as small (<200 nm) or medium/large (>200 nm). In the group of small EVs are included the exosomes and the small microvesicles, while larger microvesicles, and oncosomes belong to the medium/large group. Importantly, while some EVs are released directly via plasma membrane budding, exosomes have a different origin, from the endosomal compartment as intraluminal vesicles within the multivesicular body (MVB). Then, exosomes are released after fusion of MVB with the plasma membrane.^11^^,^^12^ However, novel classes of EVs are emerging, whose origin and function(s) remain uncertain.^17^ Currently, there are not recognized markers specific for each sub-population of EVs. The biogenesis of EVs is still under investigation, but almost all vesicles contain some class of proteins, such as tetraspanins (e.g., CD63, CD9), used as generic target molecules for EV detection and immobilization.

EVs gained attention in the last decades for their key role in cell-to-cell communication, both in physiological and pathological states. Indeed, EVs are able to deliver their bioactive payloads (e.g., DNA, RNA (mRNA, miRNA, and lncRNA), metabolites, lipids and proteins (including active enzymes)) to target cells, thus influencing their fate.^18^^,^^19^ Interestingly, target cells may be located either in proximity of the EV-donor cells, or in distant sites.^20^ For this reason, circulating EVs are recovered from almost all biofluids (e.g., blood, saliva, urine, amniotic fluid, milk, and cerebrospinal fluid), for the non-invasive discovery of novel biomarkers.^1^^,^^21^

Historically, differential ultracentrifugation is the most used method for EV purification, in some case combined with buoyant density separation for a better purification.^22^ Other approaches are based on precipitation, size exclusion chromatography,^23^ ultrafiltration^24^ and tangential flow filtration.^25^ Among these strategies, ultracentrifugation remains one of the most efficient, but it is time and labor-consuming, it requires expensive instruments, limiting their use in the clinics. On the other hand, commercial EV-isolation kits improve time efficiency. However, these kits are expensive and EVs often display low purity.^26^^,^^27^

**Figure 1:**
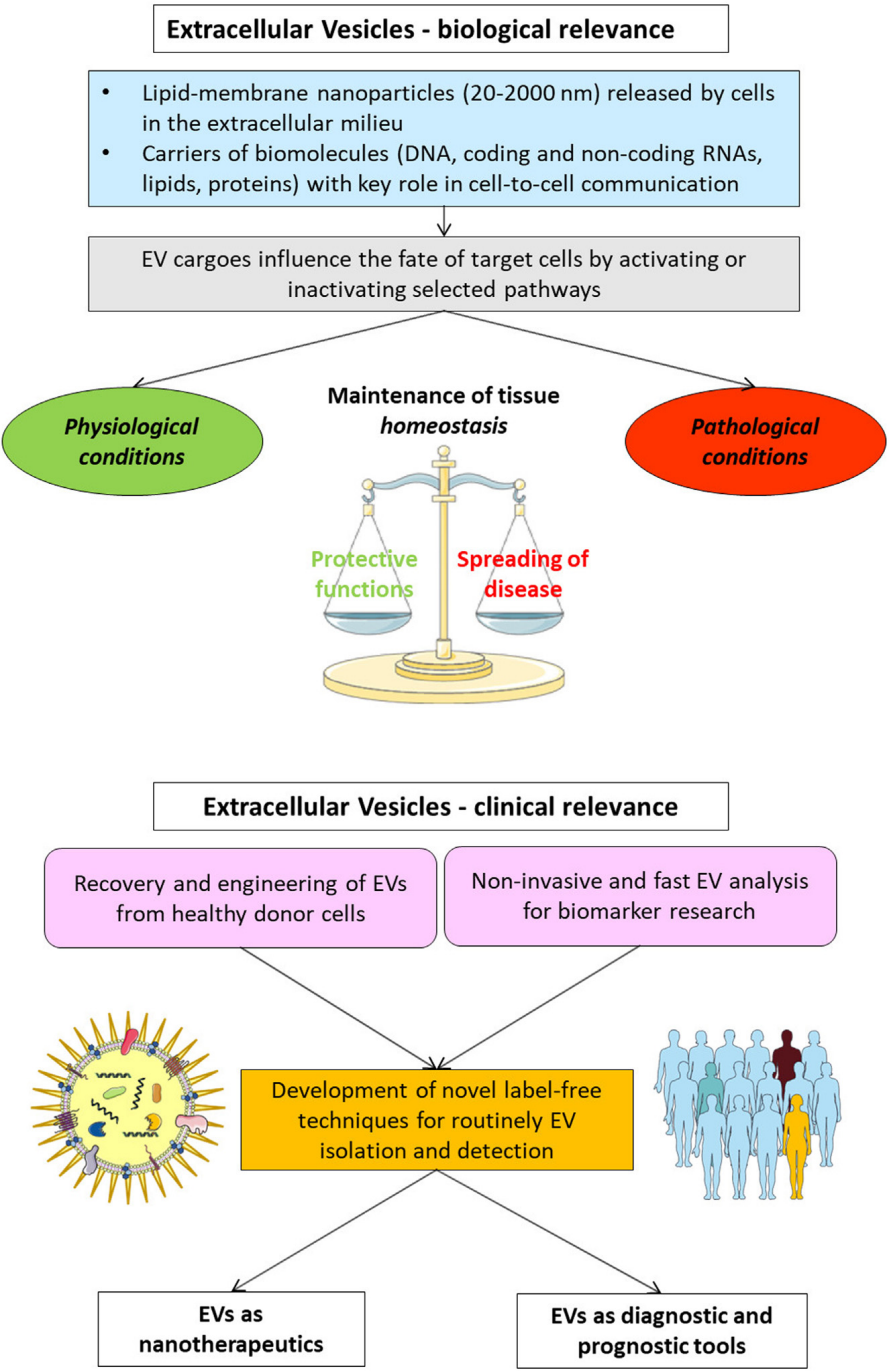
Biological Roles and Clinical Applications of extracellular vesicles Description of the emerging roles of EVs in physiological and pathological states. In physiological conditions cells exchange information via EVs that cooperate to maintain the tissue homeostasis. In pathological conditions, EVs convey negative messages to target cells thus contributing to the spreading of the pathology. Interestingly, EVs may exert also protective/reparative functions to restore the physiological state. In all cases, new methods are needed to easily isolate and analyze EVs from biological specimens. Finally, EVs may be used either as carriers to do drug delivery, or as source of diagnostic and prognostic biomarkers.

About EV detection – as suggested by the MISEV guidelines^11^^,^^12^ – common methods include, among others, nanoparticles tracking analysis (NTA),^13^ electron microscopy and high-resolution flow cytometry,^28^ which are not suitable for routine clinical applications or, in general, for low-concentrated vesicles. Indeed, it is crucial to develop novel, rapid and simple strategies to analyze the entire EV population, but also specific subpopulations, and their molecular cargoes. To this aim, label-free EV-sensing merges physical and chemical analysis, without the need for complex sample pretreatments, thereby opening the way toward on-chips and even point-of-care (PoC) low-cost analysis, at the site or nearby the patient in need (Figure 1).

## Hands-off research: Label-free detection methods

Label-free approaches can be defined as a class of methods aiming at the investigation of bioanalytes within their native and unperturbed biological conditions.^29^ After the analyte capture, the signal is obtained in a single-step, with a direct detection that avoids the use of artificial probes. This is different from conventional assays (e.g., ELISA) in which analytes are labeled to facilitate their detection. For instance, labeling can induce modifications to the molecular structure that may modify the binding affinity and specificity to interacting molecules. In addition, a label can affect the background level, as a result of non-specific interaction with other particles in the assay, finally influencing sensitivity and limit of detection.

Label-free methods solve these issues with the direct and real-time quantification of analytes by two mechanisms: (1) monitoring their selective binding to a sensor surface, from which a signal is extracted; or (2) detecting their spectroscopic fingerprint, that allows the molecular characterization of the analyte in solution. Doing so, it is possible to: (1) reduce analysis complexity and time; (2) minimize background signal; and (3) facilitate the translation to clinics laboratories.

Label-free detection methods can be classified based on the signal transduction mechanism, optical- or electrical-based. The optical-based transduction (fluorescence lifetime imaging, FLIM; surface-enhanced Raman spectroscopy, SERS; and surface plasmon resonance, SPR) are the most versatile as can be readily implemented into biomedical laboratories. FLIM and SERS allow the direct visualization of biomolecular events in solution. On the other hand, SPR leverages antibodies to capture the analyte and for the subsequent quantification by monitoring in real-time analyte-antibody binding events through SPR sensor surface, without using labeled reporter molecules in solution.

The electrical-based transduction approaches (electrochemistry, impedance, field-effect transistors) are more common in specialized laboratories, although they could be implemented in the biomedical practice given their lower cost, easiness in signal extraction and excellent sensitivity. Finally, label-free approaches may help to understand the biochemical mechanisms in which the analyzed molecules are involved, to finally facilitate the discovery of previously unbeknownst biomarkers.

## Advanced methods for label-free EV detection

**Figure 2:**
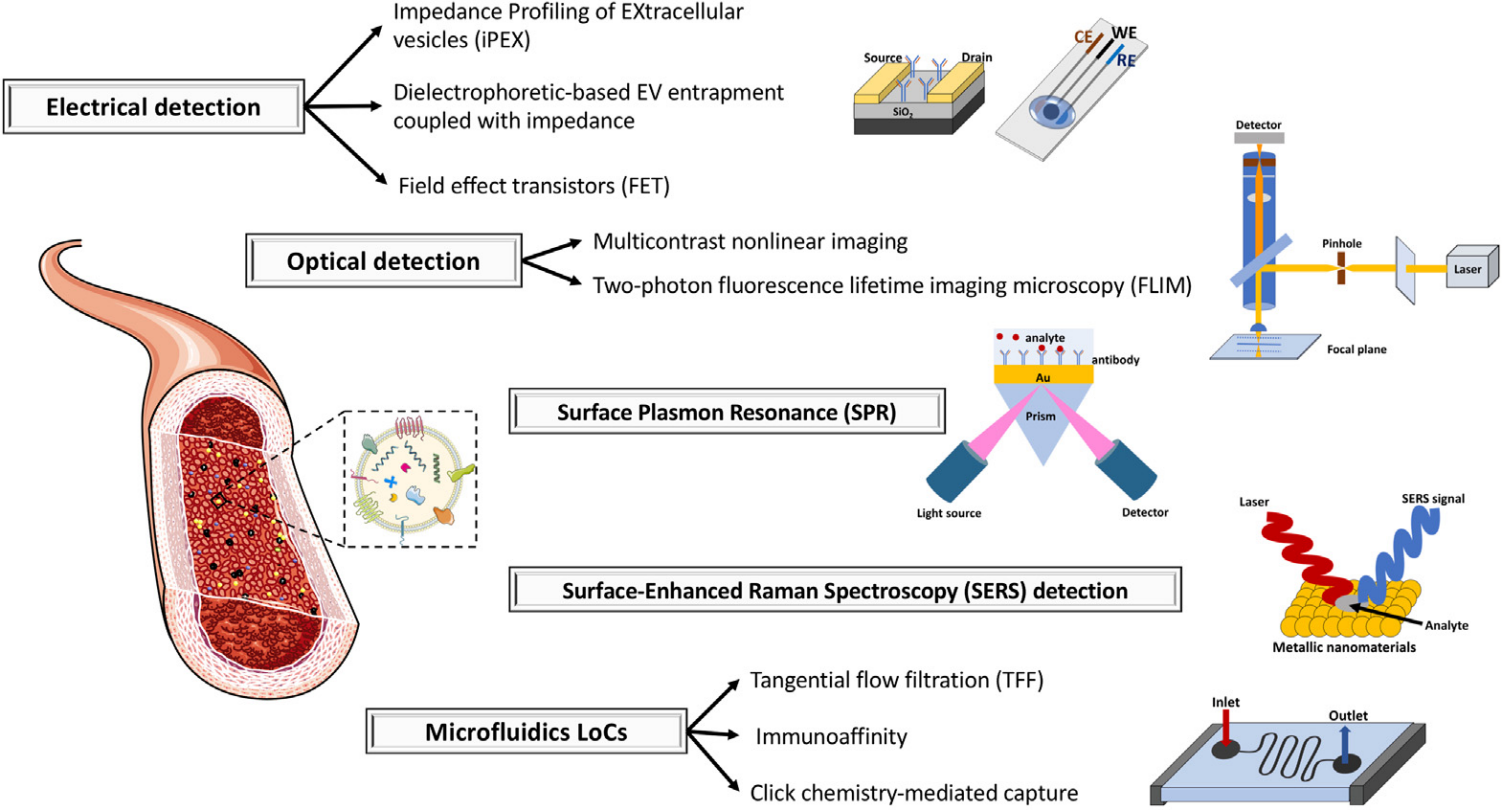
Novel label-free EV detection strategies Label-free methods on intact EVs using different analytical strategies, such as: electrical detection, optical detection, Surface Plasmon Resonance (SPR)-based detection, Surface-Enhanced Raman Spectroscopy (SERS)-based detection, and microfluidics-based lab-on-a-Chips (LoC). The implementation of LoCs via microfluidics chips permits EV analysis at low sample volume for clinical analyses. For each technique different platforms have been further developed.

Is it possible to obtain biologically relevant information from EVs at high sensitivity and in native conditions to discover new biomarkers, ultimately empowering their use in the clinical routine? The validation of novel biomarkers is the analytical challenge currently hampering the full EV exploitation in clinical settings, also considering EV cargo heterogeneity (e.g., nucleic acids, proteins etc.). The classical methods to isolate and purify EVs are not easily adaptable in the clinical routine, complying with ISO standard 15189.^30^ To overcome these limitations, a number of strategies for label-free EV detection have been optimized for direct and real-time quantification of analytes in biofluids (Figure 2). These take advantage of optical- or electrical-based signal transduction, each one having peculiar analytical features (Table 1).

**Table 1:** Pros and cons of label-free EV detection methods and their relevance for clinical settings

Label-free detection strategy	Fingerprint signal (if any)	Need for target immobilization	Discoverable EV biomarkers	Usefulness for Clinics
Fluorescence Microscopy	Fluorescence lifetime	No	Lumen biomolecules	High-resolution detection of biomarkers through microscopy
SERS	Raman specific signal	No	Lumen biomolecules and surface proteins	Development of machine learning algorithms for characterizing EV biomarkers
SPR	No	Yes	Surface proteins	Multiplexed platforms for EV capture and profiling. Validation of EV-associated biomarkers
EIS sensors	Frequency dependent signal	Yes	Lumen biomolecules and surface proteins	Rapid EV electrical fingerprint analysis
FET sensors	No	Yes	Surface proteins	High sensitivity, excellent LOD and rapid analysis

The optical detection approaches comprise fluorescence, Raman spectroscopy and surface plasmon resonance (SPR)-based methods. Intrinsically fluorescent biomolecules inside EVs, such as collagen or NAD(P)H, can be simultaneously excited by two or three photons, in a zone confined to the focal volume (∼1 fL). The two or three wavelengths used for the excitation are longer (typically in the near-infrared region) than that of the emitted photon (typically in the visible spectrum). The two-photon fluorescence excitation allows for the direct mapping of fluorescent analytes in carcinogen-induced rat mammary tumor model.^31^ NAD(P)H can be retrieved in the EV lumen also via three-photon fluorescence emission,^32^ finding that NAD(P)H concentration is higher in human breast cancer cell lines with respect to normal breast epithelial cells.^32^ Also, **fluorescence lifetime imaging microscopy** (**FLIM**) leverages the differences of the fluorescence lifetimes (below 1 and up to 100 ns) to produce images from a biological sample containing fluorescent analytes, and provides information on the environment surrounding the analyte (e.g., pH, ion concentration, viscosity). Indeed, FLIM differentiates free-from protein-bound NAD(P)H, given their different fluorescence lifetime (significantly shorter for the free form), and NAD(P)H distribution into cells vs. EVs.^33^ Ultimately, fluorescent detection permits to study the dynamics of some EV cargoes in a space- and time-dependent manner.

**Raman spectroscopy** is a non-destructive chemical analysis method that records the vibrations, able to induce a change in the polarizability of the electronic density around the molecule. Raman allows to detect either (1) components associated with the membrane (e.g., transmembrane proteins); or (2) biomolecules confined into the EV lumen (e.g., proteins, nucleic acid etc.). An improved version of Raman spectroscopy is the **Surface-enhanced Raman spectroscopy** (**SERS**), which is based on the amplification of Raman signals thanks to the adsorption of the analytes, including EVs, on compact metal nanoparticle film (defined as SERS substrate), resulting in an enhancement of the Raman-signal by a factor of 10^4^÷10^10^. SERS leverages Raman signals derived both from membrane and lumen constituents to classify EVs by multivariate data analysis or machine learning methods.^34^^,^^35^^,^^36^ Examples include fingerprint signals able to discriminate: (1) ovarian-from endometrial cancer cell-derived EVs, reaching a limit of detection (LOD) of approximately 600 EVs/mL, by using silver nanoparticles (NPs);^37^ (2) leukemia, prostate and colorectal cancer cell line-derived EVs with 97.4% accuracy, by molybdenum oxide nanoflakes;^38^ and (3) glioblastoma (GBM) cell line-derived EVs vs. noncancerous glial EVs, by metallic nanobowties.^39^ The clinical significance is demonstrated by the highly accurate fingerprint discrimination between normal and tumor cell-derived EVs.

**Surface plasmon resonance** (SPR) detection exploits electron density oscillation propagating over a thin surface of metal NPs placed onto a high-reflective index glass prism. The value of the resonance SPR angle at which electron oscillation is triggered by an incident light beam depends on the refractive index of the material near the metal surface. In turn, the resonance angle value is modified by a binding event. Indeed, EV adsorption mediated by specific ligands induces a modification of the refractive index, quantified via reflectivity measurement.^40^ SPR is an invaluable tool for EV profiling,^41^ even at single-particle level,^42^ finding important applications in biomarker discovery for cancer diagnostics. Through the binding with epidermal growth factor receptor 2 (HER2), a known breast cancer biomarker, cell line-derived HER2^+^ EVs can be captured and detected down to 8,280 EVs/μL.^43^ The discovery of biomarkers for malignant gliomas^16^ - i.e., monocarboxylate transporter 1 (MCT1) and cluster of differentiation 147 (CD147) - enabled the prompt identification of glioma-derived EVs, obtaining a linear response of the SPR biosensor at the 1.3–1,300 μg/mL concentration range. This may impact the future design of MCT1 and CD147 inhibitors as possible anticancer agents and as powerful tool for the early diagnosis of malignant transformation.

Altogether, optical detection investigates EVs based both on lumen and surface components, as valuable sources of biomarkers for clinical translation. Among the described methods, SPR likely represents the most promising for clinics, given the possibility to obtain rapid, multiplexed information for EV classification. However, optical methods are still expensive and need more user-friendly interfaces.

In alternative, electrical detection is based on EV binding on sensor surface electrodes, via antibodies or aptamers against EV membrane markers, resulting in an electrical signal that can be easily quantified. A straightforward example is constituted by electrokinetic sensing by functionalized microcapillary, to monitor the changes in streaming current upon EV binding.^44^ This sensor allowed determination of non-small-cell lung cancer and embryonic kidney cell-derived EVs through their surface markers epidermal growth factor receptor (EGFR), CD63 and CD9, with a sensitivity of ∼0.4 pM, in less than 2 h of sample incubation.^44^

An emerging label-free approach is based on **Electrical impedance spectroscopy (EIS)**, an analytical method based on the perturbation of an electrochemical system by a frequency dependent electrical signal and the subsequent recording of the electrical response. This approach allows EV sensing at the solution-electrode interface by using alternated electrical currents.^45^^,^^46^ Depending on the frequency range applied, impedance spectra may provide information about both lumen and membrane EV components. For instance, EIS has been employed in an approach defined iPEX (impedance Profiling of EXtracellular vesicles), in which an antibody against CD63, functionalized with polypyrrole on a carbon paste electrode, allowed the selective capture of GBM-derived EVs.^47^ The chip performances were demonstrated to have an EV detection range over five orders of magnitude (10°-10^6^) and a LOD of ∼500 EVs/mL. To further demonstrate the clinical validity, electrodes functionalized with GBM markers (EGFR, EGFR variant III (EGFRvIII), platelet-derived growth factor receptor alpha (PDGFRA)) were used to capture EVs from plasma samples (100 μL volume), finding that the expression of the GBM markers was higher in patients compared with healthy subjects.^47^ Another chip could also underpin the differences in terms of EVs vs. lipoproteins, a well-known contaminant when analyzing plasma-derived EVs.^48^

**Field effect transistors** (**FET**) employ an electric field to control the flow of current in a semiconductor by applying a voltage to the gate electrode. It is another strategy for electrical EV sensing, based on the response of graphene films functionalized with EV capture molecules, such as anti-CD63.^49^ The graphene surface can be functionalized to obtain a 3D morphology (e.g., carbon nanodots) which facilitates EV absorption, further enhancing the sensitivity of the system. These FET configurations allow for extremely low LOD, leading to a LOD of 100 particles/μL,^49^ or even down to 33 particles/μL.^50^ Also, a graphene FET biosensor can be integrated within a microfluidic chip (see next section), leading to EV detection at least up to 0.1 μg/mL.^51^ Here, EVs from healthy subjects led to a positive shift of the FET signal with respect to blank (PBS only).^51^

Currently, challenges linked with the expertise needed for devices’ manufacturing slow down the process of translating the actual use of electrical approaches to clinics. However, the utilization of commercial screen-printed electrochemical sensors or the functionalization of FET with EV-specific antibodies might help to overcome these issues. Additionally, manufacturing costs are decreasing over time, and the sensitivity outperforms optical detection. Finally, the possibility of integration in microfluidic chip will help the development of a new generation of EV analysis by EIS, similarly to what already done with living cells.

In summary, label-free methods allow quick and sensitive detection of EVs directly from body fluids (Table 1). Speaking of translational potential, optical methods – and in particular SPR – may represent the optimal option for efficient biomarker panel discovery. Electrical approaches are praised for the excellent analytical performances, however their use with patients is still challenging, due to issues with both detecting EVs in low-concentrated samples and lack of easy-to-use analytical platforms. As described below, these label-free methodologies may be combined with small supports based on microfluidics, which may help to improve both analytical sensitivity and system automation.^52^

## Lab-on-a-chip (LoCs): Tiny detectors for tiny vesicles

LoCs are devices that perform multiple laboratory processes into a miniaturized platform (from millimeters to a few square centimeters) by implementing microfluidics technologies. Such miniaturization allows increasing parallelization, multiplexing, analytical sensitivity along with a reduction of the sample volume (from nanoliters to picoliters). LoC systems miniaturize all the component units of an assay; hence the term “microfluidics-based LoCs”. These devices are realized by microfabrication techniques through the use of materials (e.g., metals, glass, silicon, organic polymers and polydimethylsiloxane) possessing suitable transparency, biocompatibility and flexibility.^53^^,^^54^ Indeed, microfluidics-based approaches have found several applications in disease diagnosis, prognosis and treatment.^22^

The integration of EV analytical techniques into LoCs, represents the gold standard to be achieved to get closer to patients.^55^^,^^56^ In comparison with traditional separation methods to recover EVs from large sample volumes (e.g., cell culture supernatant), microfluidics LoCs efficiently work with small-volume and low-concentrated EV samples, are highly sensitive, and show better separation yields while reducing the amount of time needed for EV isolation.^57^^,^^58^

In EV analysis, LoCs allow for the separation of solid particles dispersed in liquids, leveraging their physical-chemical parameters.^22^ In particular, microfluidics can be employed for EV isolation by passive and active technologies. In passive chips, EVs are captured without external forces via either size-exclusion,^59^^,^^60^ filtration,^61^^,^^62^^,^^63^ inertial lift force,^64^ viscoelastic flow,^65^^,^^66^ deterministic lateral displacement^67^ and immunoaffinity.^68^ Active chips are based on acoustic waves,^69^ dielectrophoretic and electrophoretic techniques^70^ and magnetic immunoaffinity methods.^71^ All these approaches were already largely discussed elsewhere.^72^^,^^73^ For instance, *Exodisc* is one of the first tabletop-sized centrifugal microfluidics system integrated with two nanofilters, to efficiently recover EVs from cell culture supernatant and patient urine samples.^74^ A more recent version, the *Exodisc-B*, allows EV isolation also from the whole blood.^62^ Both systems are already in the market and guarantee to isolate EVs in 10–40 min with high yield and purity. Advanced label-free EV detection via novel efficient platforms - high-throughput, user-friendly and cost-effective - allow to get unmodified EVs useful not only for diagnosis, but eventually for (nano)therapy.

The development of tangential flow filtration (TFF) in a microfluidic chip allowed to obtain EVs in less than 3 h, but with a preliminary purification step.^63^ Compared to conventional filtration, in TFF systems the fluid goes parallel to the filter, avoiding blockage and offering a high filtration capacity. Protein contaminants were removed (>97%), and EV recovery rate was >80%.^63^ To increase the separation efficiency, a double TFF-based microfluidic device has been recently tested with serum from liver cancer patients. The proteomics analysis on EVs demonstrated the specificity of this chip to identify proteins related to liver disease.^75^ EVs can be also separated based on their size. A novel strategy employed a continuous-flow label-free microfluidics device, combining two electrokinetic phenomena (electrothermal fluid rolls and dielectrophoresis) to isolate serum EVs with high recovery rate and purity (∼80%).^76^

Another chip based on **click chemistry** was employed for EV isolation from Ewing Sarcoma (ES) cell lines.^77^ Click chemistry is a chemical method that develops selective reactions that, by the heteroatom links (C−X−C), generate new compounds. The leucine-rich repeat and immunoglobulin-like domain-containing nogo receptor-interacting protein 1 (LINGO1) was identified as a specific ES surface marker. Antibodies against LINGO1 were used for the click chemistry-mediated EV capture chip, with high efficiency and specificity for ES-EVs, thanks to the use of anti-LINGO1 instead of anti-CD63 antibodies. Notably, vesicles maintained their integrity and biological activity after the isolation.^77^ Altogether these approaches – although in their infancy – offer an easy method for fast and sensitive EV isolation and quantification, which are critical points for EV analysis in clinics (Table 2).

**Table 2:** Microfluidics-based EV isolation methods

BIOLOGICAL CONTEXT	SOURCE	TECHNIQUE	YIELD	FLOW RATE	PURITY	Reference
General disease	Human breast adenocarcinoma cell line MCF-7 Lung adenocarcinoma cell line H1975	Size exclusion	90% (separation efficiency)	Nanosuspension between 100 nm and 1000 nm	85%	Yeo t al.^59^
Neurological diseases	Human glioblastoma astrocytoma cell line U-251 MG Human neuroblastoma cell line SY5Y LMH cell line ATCC CRL-2117	Size exclusion	47.5 ± 5.1 and 55.4 ± 4.2% for small and large EVs respectively (capture efficiency)	100 - 500 μL/min (flow rate range)	NA	Yeh et al.^60^
General disease	Adenocarcinomic human alveolar basal epithelial cells A549 Fetal Bovine Serum (FBS)	Viscoelastic flow	>80% (recovery rate)	∼100 μL (volume of sample) 200 μL/h (flow rate)	>90%	Liu et al.^66^
Bladder cancer	Urine samples	Filtration	>95% (recovery rate)	1 mL solution of EVs at 1.47 × 10^11^ particles/mL	>95% removal of protein contaminants	Woo et al.^74^
Prostate and lung cancer	Whole blood samples (healthy, prostate cancer and lung cancer patients) Plasma samples (healthy and prostate cancer patients)	Tangential flow filtration	>75% (capture efficiency from blood)	30-600 μL (volume of whole blood) 10 -200 μL (volume of plasma)	NA	Sunkara et al.^62^
General disease	Plasma samples (healthy patients)	Tangential flow filtration	>80% (recovery rate)	0.5–5 μL/min (flow rate range with optimal value 1 μL/min)	(1.18 ± 0.21) × 10^11^ particles/mg protein	Han et al.^63^
Liver cancer	Hepatic stellate normal cells LX2 and hepatoma cells HepG2 and Huh7 Human serum	Double tangential flow filtration	77.8% (recovery rate)	30 μL/min (flow rate range with highest recovery rate)	82.8%	Hua et al.^75^
General disease	Human embryonic kidney cells (HEK 293T) Rabbit serum	Electrokinetic separation	79.3% ± 2.4% (for supernatant) 75.4 ± 3.3% (for serum) (recovery rate)	(2.72 ± 0.14) × 10^6^ EV per mL (for supernatant) (2.41 ± 0.12) × 10^7^ EV per mL (for serum) (flow rate)	∼80%	Bu et al.^76^
Ewing Sarcoma	Plasma samples (prepared by spiking Ewing Sarcoma-derived EVs into plasma from a female healthy donor)	Click Chemistry Immunoaffinity	84% (capture efficiency)	100 μL (volume of sample) 0.2 mL/h (flow rate) anti-LINGO1 recognition	NA	Dong et al.^77^

Next, purified EVs can be applied onto label-free detection chips to be further characterized. Interestingly, some chips have been designed to both isolate and detect EVs, avoiding the previous steps of purification, and further supporting their use in clinical routine.^78^

Among the optical methods, a promising SERS-based chip was used to perform a retrospective study, using plasma EVs from previously diagnosed cancer patients.^79^ The EV-SERS spectra were analyzed by artificial intelligence (AI) algorithms, and six early-stage cancer types were identified with a diagnostic sensitivity and specificity >90%.^79^ The system is low-cost, since no additional reagents are required for the analysis, and small sample volumes can be used to obtain a suitable number of EVs for the analysis. However, the chip needs already purified EVs, since contaminant molecules may interfere with the SERS signal detection. Additionally, even with a high number of training samples, EV-SERS-AI was yet unable to discriminate EVs from benign vs. malignant tumors, limiting its current use as diagnostic tool.^79^

Another optical label-free detection system is the SPR. An interesting SPR-based chip was designed to capture HER2^+^ vesicles, a potential biomarker for breast cancer, since HER2 levels are consistent between tumor tissues and tumor-derived EVs.^80^^,^^81^^,^^82^ However, SPR-based biosensors face some issues working with serum-derived EVs: (1) the small size of EVs results in a low signal and, as a consequence, a signal amplifier is required; and (2) serum contaminant proteins are responsible for false positive signals.^83^ Then, SPR was improved by using a strategy called tyramine signal amplification.^84^ First, gold-NPs were conjugated with tyramine (Au-NPs-Ty), then the gold surface of the chip was functionalized with HER2 aptamers for binding EVs, plus special DNA sequences (G-quadruplex). Once EVs were captured by HER2 aptamers, the G-quadruplex DNA mediated the recognition of the lipids in the EV membranes, finally enhancing the SPR signal. This strategy overcomes the limitation of classical SPR approach, thanks to the dual recognition of HER2 and EV lipids, avoiding the interference from contaminants in the samples.^84^ Again, although very promising, this system needs isolated EVs prior the analysis.

SPR was further implemented with a digital EV analyzer software, for the automatic EV analysis and profiling.^85^ A panel of aptamers was used to bind EVs, including CD63, epithelial cell adhesion molecule (EpCAM), HER2, prostate-specific membrane antigen (PSMA) and protein tyrosine kinase 7 (PTK7). The subsequent analysis discriminated EVs of different origin with an accuracy of 73%, opening up the way to robust clinical assays.^85^

Among the label-free electrical-based approaches, a novel microelectronic EIS chip was developed to detect and characterize small EVs from cancer cell line supernatants.^78^ The device included an insulator-based dielectrophoretic (iDEP) module to isolate EVs, together with the EIS micro-electrodes for the detection. The system evaluated unique dielectric properties of the vesicles, and was able to distinguish EVs from different cell types in 15 min, characterizing the presence of both membrane and lumen components.^78^ Despite these interesting properties, including the possibility to separate and detect EVs in a single chip, further studies are needed to better identify distinct EV molecular cargoes, for the use in a clinical setting.

As mentioned, FET technology may be associated to microfluidics. In particular, different FET biosensors, opportunely conjugated with anti-CD63 antibodies, were shown to selectively detect EVs in a label-free setup, with a remarkable LOD down to 33 EVs/μL.^49^^,^^50^^,^^51^ Again, these systems require already purified EVs before the loading in the microfluidic channel. Although promising, they need more implementations for clinical applications, considering the limited capacity in terms of EV classification.

The great number of studies describing LoCs for EV analysis reflect the direction that the field is following. Indeed, thanks to the possibility to isolate and detect low levels of EVs in biological samples, the easy sample handling and the lesser time for EV analysis, compared to classical technologies, make the microfluidics-based approaches promising tools for translation in clinics. SPR-chips allow multiplexed label-free detection with the possibility to implement clinical validation. On the other hand, FET biosensors possess better analytical features in terms of LOD and sensitivity, but they struggle to distinguish EVs from different origins. Other limitations need to be overcome, such as the lack of standardized protocols. Additionally, clinical validation through large-scale studies is still necessary. The research is progressing faster to corroborate the reliability of LoC approaches and choose them for future drug discovery/development, pharmacokinetic evaluations and toxicity screenings, or to introduce them in clinical routine for diagnostic and/or prognostic applications.

## Clinical applications

The search for reliable biomarkers of diagnosis, prognosis and response to therapies - possibly in a non-invasive way - is highly required in several diseases. EVs, with their heterogeneous range of biological cargoes (DNA, coding and non-coding RNAs, proteins, metabolites, lipids), positively or negatively affect the fate of target cells, and thus may serve as valuable sources of biomarkers, with potential translational application. Also, EVs recovered from clinically relevant sources (e.g., stem cells) may be used as innovative nanotherapeutics - per se or opportunely engineered - to deliver specific molecules at the target sites.

Label-free EV detection methods are suitable for the analysis of EVs from several biological matrices (e.g., blood, urine, etc.), without the necessity of complicated purification steps. Detecting EVs in their native conditions requires minimal sample preparation, hence saving time. Also, label-free approaches bear the advantage to preserve EV structural integrity, reducing artifacts or biases potentially linked with labeling protocols. Indeed, label-free recovered EVs are more suitable to be used as nanotherapeutics. However, although label-free techniques provide accurate information on EV size, concentration and cellular origin, they may lack details about the EV molecular cargoe heterogeneity, if not complemented with other approaches (such as mass spectrometry, proteomics, genomics). This lowers their potential use as multicomponent predictive biomarker system.

Lab-on-a-chips (LoCs) based on microfluidics ease isolation and detection of EVs from different biological matrices. When used in combination with specific capture techniques (e.g., immunoaffinity), may become powerful platforms granting the possibility to apply EV analysis to precision medicine in the near future. An efficient platform for EV analysis needs to be streamlined, from sample preparation to EV isolation, detection and quantification. Also, it should be user-friendly, cost-effective and applicable to clinical settings. Current unmet needs for using LoCs as platforms for EV study in patients may include the lack of standardized protocols and user-friendly interfaces. Furthermore, clinical validation through large-scale studies is still necessary. Also, for LoC-based techniques applied to EV studies, small-volume analyses might be a current critical bottleneck which yet restrains the effective translation from bench to bedside. A reduced starting volume of biological matrices may increment the background noise, especially in case of low-abundant EV subpopulations. Nonetheless, the potential of these strategies for EV analysis can be measured by the increasing number of companies offering services for EV molecular analysis and for the development of EV-based therapeutic applications. On-chips based platforms for EV analysis are already in the market as clinical diagnostics for PSA-independent prostate cancer assays, namely the ExoDx Prostate IntelliScore (EPI)^86^ and the miR SentinelTM PCC4 assays.^87^ They are both based on established RNA biomarkers present in urine-derived EVs, more specifically three genes as urine EV messenger RNA (mRNAs) signature for the former, and a panel of small non-coding RNAs (miRNAs and snoRNAs) for the latter. Also, several clinical trials are currently ongoing to evaluate EVs as biomarkers, and microfluidics devices are employed in some studies, supporting the importance of tiny detectors for small and low-abundant EVs.

Importantly, coupling LoCs with label-free detection strategies may enable multiplexed biomarker discovery and subsequent clinical validation, providing the pillars for future EV-based low invasive diagnostics screenings, and their further commercialization.

## Concluding remarks and future directions

In the last decade, EV research evolved at fast pace, leading to the discovery of their key role in cell-to-cell communication, and, additionally, to the characterization of their potential application as biomarkers/nanotherapeutics in pathological settings. EV-associated molecules may provide a novel layer of investigation for the development of a multicomponent predictive biomarker system. Indeed, circulating EVs are stable in biofluids, and protect their cargoes from degradation. Moreover, once recovered, EVs may be engineered to contain therapeutic molecules for treating patients. The use of own EVs would allow a more specific response, with limited side effects.^79^

However, several questions remain to be solved, starting from the limited knowledge currently available about the biogenesis of different EV sub-types. Different vesicles may shuttle distinct molecular payloads, with a possible consequent theragnostic diversity. New label-free detection approaches need to deal with the lack of clear markers able to distinguish EVs with specific mechanisms of biogenesis. In addition, these methods have to minimize the potential impact on the quality and/or the quantity of EV-derived molecules (such as impurities and EV aggregation), and thus on their informative potential.

Also, the recovery of EVs from specific cell origin would better predict the patient’s clinical outcome for a personalized therapy, but it is still debated how the new label-free methods can handle the background noise from the EVs secreted by virtually all the cells of the body. Possibly, they may help to identify novel surface markers able to discriminate EVs from different donor cells, to focus on specific body districts. In this context, the possibility of translating the detection methods from *in vitro* settings into the clinical practice is of pivotal importance. Indeed, the EV-derived candidate molecules from preliminary studies need to survive to the clinical screening in larger cohorts of patients, where the population diversity greatly contributes to variability.

These aspects underpin the development of label-free and low-sample consumption analyses, for a standardized and rapid use with patients’ specimens. Advanced fluorescence methods allow for the high-resolution detection of EVs under physiological or pathological conditions. Conversely, SERS assays couple high sensitivity with a molecular fingerprint analysis. SPR approaches have the potential to become the best option for efficient biomarker panel discovery. EV analysis through electrical-based detection show immense versatility, albeit being mainly developed by specialized laboratories, with limited clinical use, so far. AI algorithms will further support the prediction power of these approaches.

Microfluidics chips are being integrated with label-free detection for routine screening of patient-derived EVs.^88^ However, many of these novel microfluidics-based LoCs should be improved for “real-life” EV testing (i.e., vesicles from different biofluids). Also, the operative protocols need to be further simplified to make on-chips platforms for EV analysis user-friendly tools in the hands of clinicians. Indeed, the study of EV cargoes from different clinical cohorts would generate predictive panels of biomarkers eventually able to discriminate between different types/stages of diseases, with important implication for early diagnosis.

Future research is needed to fulfill the synergy between EV label-free detection with multiplexed signal analysis, and to give EVs the chance to enter in the clinics. Biologists, chemists, physicists and clinicians need to work closely to reduce the distance between different expertise and to finally obtain efficient platforms working in a context of clinical routine.
